# A Biologically Inspired Neural Network Model to Gain Insight Into the Mechanisms of Post-Traumatic Stress Disorder and Eye Movement Desensitization and Reprocessing Therapy

**DOI:** 10.3389/fpsyg.2022.944838

**Published:** 2022-07-13

**Authors:** Andrea Mattera, Alessia Cavallo, Giovanni Granato, Gianluca Baldassarre, Marco Pagani

**Affiliations:** Institute of Cognitive Sciences and Technologies, National Research Council, Rome, Italy

**Keywords:** post-traumatic stress disorder, eye movement desensitization and reprocessing therapy, prolonged exposure, computational modeling, amygdala

## Abstract

Eye movement desensitization and reprocessing (EMDR) therapy is a well-established therapeutic method to treat post-traumatic stress disorder (PTSD). However, how EMDR exerts its therapeutic action has been studied in many types of research but still needs to be completely understood. This is in part due to limited knowledge of the neurobiological mechanisms underlying EMDR, and in part to our incomplete understanding of PTSD. In order to model PTSD, we used a biologically inspired computational model based on firing rate units, encompassing the cortex, hippocampus, and amygdala. Through the modulation of its parameters, we fitted real data from patients treated with EMDR or classical exposure therapy. This allowed us to gain insights into PTSD mechanisms and to investigate how EMDR achieves trauma remission.

## 1. Introduction

Post-traumatic stress disorder (PTSD) is a maladaptive reaction to traumatic events characterized by American Psychiatric Association (2013): (a) intrusiveness of distressing memories of the traumatic event, that occur in response to reminder cues of the trauma; (b) avoidance of the trauma-associated cues; (c) negative alterations in cognitions and mood; (d) hyperarousal. Many studies found that the activation of the amygdala during the exposure to trauma reminders or fearful stimuli is significantly correlated with the severity of PTSD symptoms (Rauch et al., 2000; Pissiota et al., 2002; Fredrikson and Furmark, 2003; Shin et al., 2004; Protopopescu et al., 2005; Ganzel et al., 2007; Brohawn et al., 2010; Jacques et al., 2011; McLaughlin et al., 2014; Neumeister et al., 2017; Stevens et al., 2017). Also, the reduction of the amygdala activation after the treatment correlates with the success of psychotherapy in attenuating the symptoms (Peres et al., 2011; Thomaes et al., 2014; King et al., 2016). Moreover, patients suffering from PTSD show reduced recruitment of the brain areas involved in emotion regulation, such as the ventromedial prefrontal cortex (vmPFC) and the dorsolateral prefrontal cortex (dlPFC), when facing cues associated with trauma (Rauch et al., 2006; Liberzon and Sripada, 2007; Kasai et al., 2008; Milad et al., 2009; Shin and Liberzon, 2010).

Post-traumatic stress disorder does not occur in all individuals that experience trauma, suggesting that specific susceptibility factors determine if the disorder will develop or not (Alisic et al., 2014; Musazzi et al., 2018). For instance, during the extinction of Pavlovian fear conditioning, patients with PTSD reveal hypoactivation of the vmPFC, compared to healthy controls (Milad et al., 2009; Rougemont-Bücking et al., 2011), although it is not known whether this alteration has to be considered a cause or a consequence of the trauma (Yehuda, 1999; Kasai et al., 2008; Sun et al., 2013; Miller et al., 2017; Alexander et al., 2020).

Prolonged exposure (PE) to threatening stimuli and eye movement desensitization and reprocessing (EMDR) are first line therapeutic strategies for PTSD (World Health Organization, 2013; Schnyder et al., 2015; Cusack et al., 2016; Gainer et al., 2020). PE consists of several sessions of exposure to the trauma-related stimuli in a safe context. The exposure can also be imaginal. In the latter case, the therapist asks the patient to relive the traumatic experience as it was happening at that precise moment (Foa and Rothbaum, 2001). When the distress arising from physical or imaginal exposure is too high, PE can be paired with anxiety reduction techniques, such as slow breathing or mindfulness (Brewer, 2001; Frye and Spates, 2012).

It has been suggested that vmPFC activation during exposure and the resulting downregulation of the amygdala are key factors of PE therapy (Stojek et al., 2018). It is worth noting that patients with PTSD in whom the vmPFC is more active during emotional conflict tasks benefit from a greater symptoms reduction after PE (Fonzo et al., 2017). This is in agreement with the proposed role of the vmPFC in discriminating safety signals and inhibiting the amygdala during fear extinction (Phelps et al., 2004; Schiller et al., 2008; Feng et al., 2016; Fullana et al., 2016; Via et al., 2018; Tashjian et al., 2021). Murine studies and simulations confirm this picture. Indeed, the homologous brain region in mice—the infralimbic cortex—is progressively recruited during the exposure to no longer threatening conditioned stimuli and promotes synaptic plasticity and fear extinction in the amygdala through long range projections (Garcia et al., 1999; Milad and Quirk, 2002; Cho et al., 2013; Moustafa et al., 2013; Senn et al., 2014; Do-Monte et al., 2015a; Mattera et al., 2020). Even though the vmPFC is believed to fire in response to safe stimuli and context in an automatic way (Gyurak et al., 2011), it has been shown that it can also be recruited endogenously and actively, for example through exercises of mindfulness (Zeidan et al., 2014).

The mechanisms of action of EMDR have been widely debated (Lohr et al., 1998; Herbert et al., 2000; Rogers and Silver, 2002; Lee et al., 2006; Pagani et al., 2017; de Voogd et al., 2018; Landin-Romero et al., 2018; Baek et al., 2019; Holmes, 2019). During EMDR sessions, the patient is instructed to keep the most disturbing image, the negative feelings, beliefs, emotions, and the body sensations associated with the trauma in mind, while following an alternating bilateral stimulation (e.g., right-left hand movements, bilateral fingers tapping, or bilateral auditory stimuli) from the therapist (Gainer et al., 2020). An important characteristic of this therapy is that patients show a faster symptom improvement, usually in 6–8 sessions (Power et al., 2002; Shapiro, 2014; Proudlock and Peris, 2020), compared to the PE recovery that lasts on average 12 sessions (Foa and Rothbaum, 2001; Banducci, 2021).

The neurobiological correlates of EMDR have been investigated in real time, with millisecond resolution, through electroencephalography (EEG) recorded during the whole session. Notably, the bilateral stimulation induces an immediate synchronization of all cortical areas in the delta band (1–4 Hz; Harper et al., 2009; Pagani et al., 2011, 2012). On the basis of these results, it has been proposed that slow waves arising during EMDR enact a sleep-like mechanism of memory consolidation (Pagani et al., 2017). Indeed, during sleep, recent memory traces are reactivated simultaneously in the hippocampus and the slowly oscillating sensory and prefrontal cortices (Sirota et al., 2003; Ji and Wilson, 2007; Peyrache et al., 2009; Helfrich et al., 2019). It is thought that this process is the basis of a hippocampus-to-cortex transfer, where episodic memories can be integrated into the existing cognitive schemes (Sirota et al., 2003; Mölle and Born, 2009; Diekelmann and Born, 2010). Experimental disruption of memory reactivation or impairment of slow waves impinges memory consolidation (Miyamoto et al., 2016, 2017). On the other hand, a stimulation mimicking slow waves induces long term potentiation in neocortical neurons (Chauvette et al., 2012; Sandler et al., 2016) and enhances memory retention (Miyamoto et al., 2016). Theoretical investigations indicate that slow oscillations boost synaptic plasticity and associative learning between cortical areas (Wei et al., 2016; Capone et al., 2019; Golosio et al., 2021).

The adaptive information processing (AIP) model of Shapiro proposes that traumatic memories are not integrated into the existing memory networks and remain stored in a maladaptive form (Solomon and Shapiro, 2008). The slow waves-promoting effect of EMDR suggests that therapy would promote the transfer of memories from the hippocampus-amygdala complex to the cortex, where they can be integrated into the associative cortical networks; this would allow to process the traumatic memory in an adaptive form, leaving the cognitive aspects of the memory intact, while erasing the associated emotional trace (Stickgold, 2002; Pagani et al., 2017). As in Shapiro's AIP model, the cortical transfer would help to make sense of the trauma by connecting the memory with the previously acquired cognitive schemes (Solomon and Shapiro, 2008).

Besides slow oscillations, another important insight into the mechanisms of EMDR comes from the *Working Memory Hypothesis* (de Voogd et al., 2018; Landin-Romero et al., 2018). This hypothesis suggests that tasks engaging in working memory reduce traumatic memory intrusion and downregulate the amygdala (Holmes et al., 2009; Qin et al., 2009; Schweizer et al., 2013; James et al., 2015; Iyadurai et al., 2018). It has been observed that bilateral eye movements during the presentation of conditioned stimuli previously associated with an electric shock activate the dlPFC and inhibit the amygdala, similarly to a working memory task. Moreover, it has been shown that, contrary to PE therapy, the vmPFC is deactivated by bilateral eye movement during the processing of traumatic memories (de Voogd et al., 2018). This evidence suggests that PE and EMDR exert their therapeutic action through the recruitment of different subsets of the PFC areas possibly having different efficacy in fear inhibition.

As many brain areas are involved, the integration of the phenomena involved in PTSD and its therapies in a whole coherent framework posits a challenge. Moreover, we still lack models of PTSD able to account for the whole complexity of the disease. On one hand, imaging research in patients often has substantial problems with resolution and reliability (Nord et al., 2017; Kredlow et al., 2022), and does not allow insight into the actual computations exerted by the investigated brain area (Logothetis, 2008). On the other hand, murine models permit a finer resolution and manipulation of the circuits and neuronal populations, but often fail to recapitulate the actual characteristics of the disorder. In particular, while Pavlovian fear conditioning—the most used PTSD-mimicking protocol in mice—reproduces some aspects of PTSD (Mahan and Ressler, 2012; Verbitsky et al., 2020), the continuous exposure to the conditioned stimulus no longer associated with the noxious stimulus causes the extinction of conditioned fear after some trials (Mattera et al., 2020), while PTSD symptoms are particularly resistant to extinction and can last decades (Morgan et al., 2003; Chapman et al., 2012; Careaga et al., 2016). Moreover, although humans can extinguish Pavlovian fear with exposure (Kalisch et al., 2006), in the case of PTSD the exposure to trauma reminders outside a therapeutic context can worsen the symptoms (Eysenck, 1982; Hassija and Gray, 2007).

Computational models can be a tool to face the outlined complex problem through the integration of disparate experimental information in the same theoretical framework (Eliasmith and Trujillo, 2014; Nair et al., 2016). Models are constrained by experimental data to gain biological plausibility and, at the same time, can incorporate new hypotheses to be tested. The emergent properties of the model produce insights into the mechanisms possibly underlying the studied phenomena, and allow predictions that are testable in future experiments, grounded on the hypotheses incorporated in the model and the data used to constraint it (Shen and McNaughton, 1996; Nair et al., 2016). Here, we used a biologically inspired neural network to model PTSD, and the effects of PE and EMDR, to verify the computational plausibility and coherence of the proposed mechanisms of action. In particular, to reproduce the overall effect of EMDR, we adjusted two parameters representing the inhibitory activity of PFC on the amygdala and the enhanced cortical learning rate induced by the slow waves. Moreover, the simulations allowed us to reproduce experimental data and gain insights into their neurobiological implications.

The article is organized as follows. First, we describe the PTSD model and its biological underpinnings (Section 2). Then, we test its robustness through the reproduction of different PTSD related phenomena (Sections 3.1, 3.2). Next, we reproduce the proposed mechanisms underlying PE and EMDR to simulate data from patients (Nijdam et al., 2012) and derive information regarding the different time courses of the two therapies (Sections 3.3, 3.4). Finally, we discuss the results of the simulations and their contribution to the understanding of PTSD (Section 4.1) and EMDR (Section 4.2).

## 2. Materials and Methods

### 2.1. Neural Units

**Figure 1:**
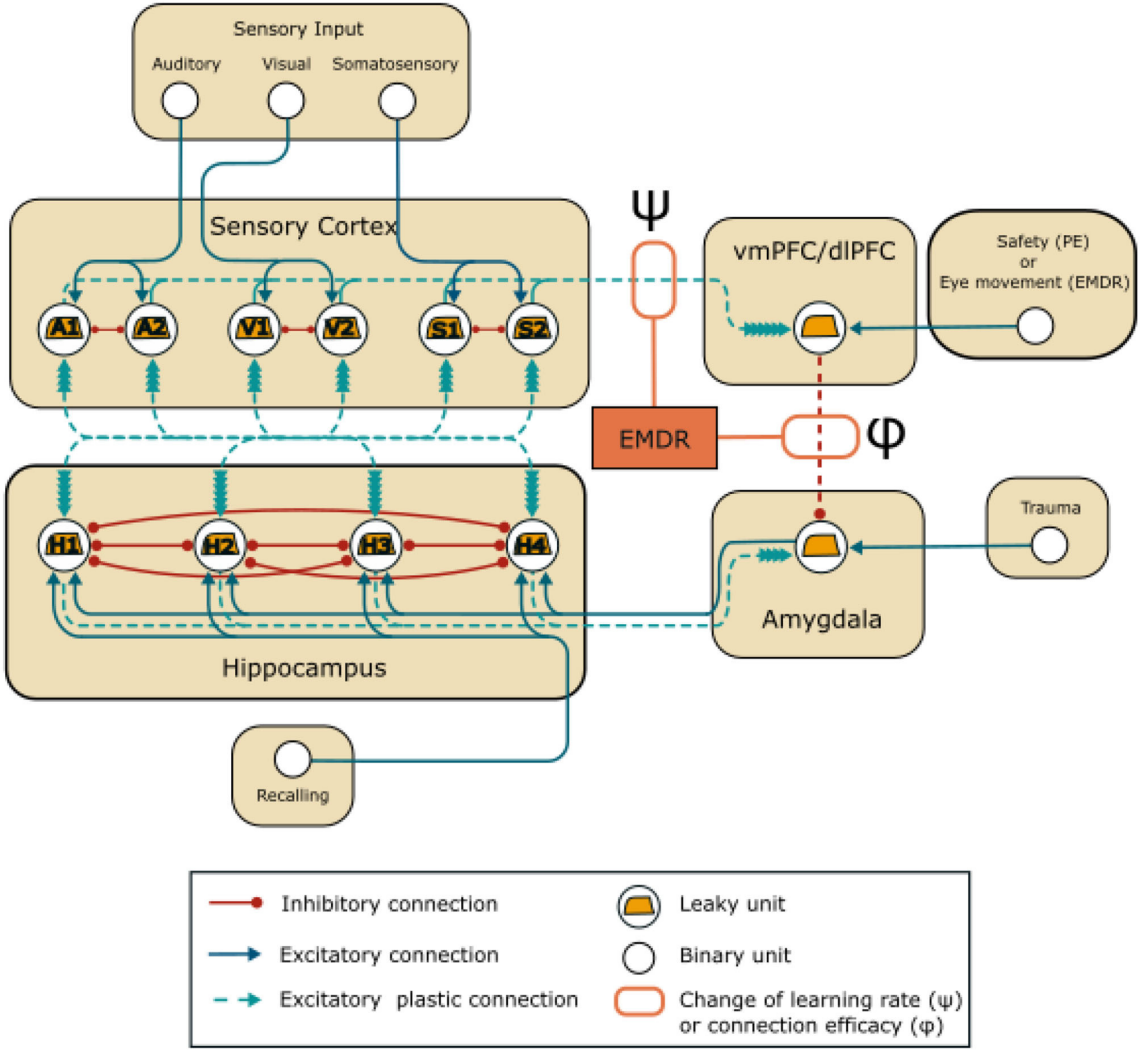
Model architecture: units and connections of the model. EMDR acts on the strength of the inhibition from the PFC (vmPFC in PE and dlPFC in EMDR) to the amygdala through the parameter ϕ, and on the learning rate of the connections between the sensory units and the PFC through the parameter ψ.

The neural units forming the model are *leaky units* (Dayan and Abbott, 2001), each representing the activity of a population of neurons with the same electrophysiological properties (Moustafa et al., 2013; Carrere and Alexandre, 2015; Mannella et al., 2016; Mattera et al., 2020). These units are characterized by membrane potential and a firing rate. In leaky units the change of the unit potential in time, ${\overset{∙}{V}}_{post}$, is represented with the following differential equation, approximated with the Euler method:


(1) $$\begin{array}{r} \tau \cdot {\overset{∙}{V}}_{post}=-V_{post}+I+\underset{pre}{\sum }\left(w_{post,pres}\cdot F_{pre}\right) \end{array}$$

where τ is the time constant, *I* is the external input to the unit (representing the activation of the sensory cortices, the recalling signal to the hippocampus, the safety signal to the vmPFC in PE or the eye movement in EMDR, the trauma to the amygdala; refer to Figure 1), *w*_*post, pre*_ is the weight of the connection between the pre- and post-synaptic unit, *F*_*pre*_ is the firing rate of the presynaptic units. The firing rate of the unit, *F*_*post*_, was calculated with the hyperbolic tangent function *tanh*(*x*):


(2) $$\begin{array}{r} F\left(V_{post}\right)={\left[tanh\left(V_{post}-\theta \right)\right]}^{+} \end{array}$$

where θ is the firing threshold and [*x*]^+^ the positive function ([*x*]^+^ = *x* if *x*≥0, and [*x*]^+^ = 0 if *x* <0). The connection weights at the beginning of the simulation, as well as the firing thresholds of the neurons, are listed in the Supplementary Table 1.

### 2.2. Model Connectomics and Its Biological Underpinnings

The model involves four areas (Figure 1), representing the sensory cortices, the hippocampus, the amygdala, and the PFC. The hippocampal-amygdala network is organized as a bidirectional associative network, inspired by Alvarez and Squire (1994). It has been theorized (Tryon, 1998), but never shown with simulations (Radell et al., 2017) that this kind of neural network would recapitulate the symptoms of PTSD. Associative networks are indeed capable of pattern completion, i.e., the ability to reconstruct a whole pattern from a single cue (Alvarez and Squire, 1994; Tryon, 1998), and this is a characteristic of the intrusive memory flashbacks that follow a trauma-reminder cue (Brewin, 2015; Ehlers, 2015).

The hippocampus receives input from unimodal and multimodal sensory cortices, that can converge in the same hippocampal population (Fried et al., 1997; Quiroga et al., 2009). Lateral inhibition and sparse connectivity at the dentate gyrus level ensure the separation of information, thus strongly segregated patterns of contextual input from sensory cortices activate non-overlapping hippocampal populations (Norman and O'Reilly, 2003; Rolls, 2013; Espinoza et al., 2018). In the model, these characteristics are captured by a winner-take-all architecture (Alvarez and Squire, 1994). The initial weights from the neuronal units in the sensory cortices to the hippocampal units and from the hippocampus to the sensory cortices are extracted randomly from a uniform distribution (Supplementary Table 1). This allows a different activation of the hippocampal units for each possible input pattern. A strong lateral inhibition between the hippocampal units ensures that only one hippocampal unit wins the competition and fires persistently in response to a sensory pattern. On the other hand, each sensory modality is represented by two mutually inhibiting units (units A1 and A2 represent the auditory cortex, S1 and S2 the somatosensory cortex, and V1 and V2 the visual cortex). Importantly, the sensory cortices in Figure 1 represent both primary cortices and also higher level unimodal cortices. Differently from Alvarez and Squire (1994), which used simplified firing rate neurons, we did not implement lateral excitatory connections between the sensory cortices. This has been done to avoid a perpetual activation induced by an excessive recurrent excitation due to the more realistic dynamic nature of the leaky neurons used here. This implies that, in our model implementation, the memory trace is not transferred form the hippocampus to the cortex, as in Alvarez and Squire (1994). Since obtaining this feature with the more realistic neurons used here would have been overly complicated, we decided to not consider this aspect as it goes beyond the scope of the research objective pursued here.

It has been shown that recalling a particular experience reactivates the same hippocampal population that fired at the moment of the actual experience (Rolls and Xiang, 2006; Gelbard-Sagiv et al., 2008). Moreover, this internally generated hippocampal reactivation is capable of reinstating the original cortical representations (Wheeler et al., 2000; Tanaka et al., 2014). Our model reproduces this property through an associative learning process taking place at the level of the reciprocals connections between the sensory cortices and the hippocampus (Alvarez and Squire, 1994; Rolls, 2007; Schwindel and McNaughton, 2011). This learning process is driven by the Bienenstock-Cooper-Munro (BCM) learning rule (refer to Section 2.3).

Hippocampus and amygdala are reciprocally connected (Figure 1), as shown by anatomical investigation (Pitkänen et al., 2000). In murine, hippocampal projections directly target amygdala fear neurons, i.e., the neuronal population that becomes active after the establishment of fear conditioning (Herry et al., 2008). In particular, the hippocampus conveys contextual information to the amygdala (Phillips and LeDoux, 1992; Kim and Cho, 2017) through synaptic projections that are potentiated by fear learning (Kim and Cho, 2020). In humans, after contextual fear conditioning, the context associated with an electric shock evokes a coupled activation in both hippocampus and amygdala, as revealed by imaging studies (Alvarez et al., 2008; Marschner et al., 2008; Lang et al., 2009; Baeuchl et al., 2015).

Finally, the amygdala is under the inhibitory control of the PFC. vmPFC and dlPFC are respectively engaged in PE (Phelps et al., 2004; Schiller et al., 2008; Feng et al., 2016; Fullana et al., 2016; Via et al., 2018; Tashjian et al., 2021) and in EMDR therapy (de Voogd et al., 2018). In the model (Figure 1), the PFC unit represents vmPFC or dlPFC, depending on the therapy that is being simulated and receives plastic inputs from the unimodal sensory cortices. These should not be intended to represent direct anatomical projection, but functional indirect connections that are modulated by learning (Bhanji et al., 2019; Ginty et al., 2019). In Figure 1, and throughout the article, the weight of the connection between the PFC and amygdala is indicated by ϕ. The initial value of ϕ (Sections 3.1, 3.2, 3.3) was set to −1 (Supplementary Table 1). Subsequently (Section 3.4), it was modified according to the results of the grid search algorithm that was used to tune the parameters: this was done to reflect a possible difference in the amygdala inhibition efficacy of vmPFC and dlPFC. Indeed, we reasoned that if EMDR recruits different PFC areas compared to PE, this would be reflected in a different total efficacy of amygdala inhibition (the parameter ϕ) by the whole vmPFC/dlPFC compound.

### 2.3. Plasticity Equations and Their Biological Underpinnings

The weights of the plastic connections (refer to Figure 1) are updated according to a simplified BCM learning rule (Bienenstock et al., 1982):


(3) $$\begin{array}{r} \Delta W_{post,pre}=\alpha \cdot \left(F_{post}-\rho \right)\cdot F_{pre} \end{array}$$

where α is the learning rate, ρ is the plasticity threshold (Supplementary Table 1), F_post_ and F_pre_ are the post- and pre-synaptic firing rates. Following this rule, a connection undergoes long-term potentiation (LTP) or long-term depression (LTD) if the firing of the post-synaptic unit is respectively above or below the threshold ρ. The weights are clipped within a (*Wmax, Wmin*) range (Supplementary Table 1).

An influential paradigm states that the cortex is a slow learner, while the subcortical structures are fast learners. This would allow the gradual integration of episodic memories with previous knowledge through slow consolidation, and favor the integration rather than interference between old and newly acquired memories (McClelland et al., 1995; Frankland and Bontempi, 2005). This theory is supported by experimental findings concerning the temporal dynamics of memory transfer from subcortical to neocortical zones (Zola-Morgan and Squire, 1990; Kim and Fanselow, 1992; Quinn et al., 2008; Do-Monte et al., 2015b), by the well-known difficulty of producing synaptic plasticity in neocortical slices compared to hippocampal ones (Bear et al., 1992; Bear and Kirkwood, 1993), and by computational models (Alvarez and Squire, 1994; Hattori, 2014). Even though some cases of remarkably fast cortical engram formation have been found (Kitamura et al., 2017; Brodt et al., 2018), it has been shown that rapid cortical learning requires that the new information is consistent with previously acquired cognitive schemes (Tse et al., 2007, 2011; Squire et al., 2015; Kumaran et al., 2016).

We incorporated slow cortical learning into the equation driving synaptic plasticity in our model. In particular, the learning rate of the connections between the sensory cortex and the dlPFC/vmPFC is 10 times slower. We choose this value accordingly to Alvarez and Squire (1994), where an order of magnitude of difference in cortico-cortical learning rate compared to the hippocampal learning rate allowed the reproduction of the gradual hippocampus-cortex information transfer found in experiments. The model also reproduces the plasticity-promoting effect of slow waves induced by bilateral stimulation in EMDR (Chauvette et al., 2012; Sandler et al., 2016; refer to Section 1). In particular, we simulated this effect by modifying the strength of the plasticity acting on the cortical connections through the parameter ψ (refer to Figure 1):


(4) $$\begin{array}{r} \Delta W_{cortex}=\alpha \cdot \psi \cdot \left(F_{post}-\rho \right)\cdot F_{pre} \end{array}$$

### 2.4. Simulation Protocols

Input units to the model (Figure 1) are binary units that can be in two different states, “off” and “on”. Only in the “on” state do they send an input equal to 1 to the postsynaptic unit.

The sensory cortex units are organized in mutually inhibiting couples: A1- A2, S1-S2, V1-V2, representing neuronal populations in the auditory cortices, somatosensory cortices, and visual cortices. Among the 8 possible patterns of stimulation, we have chosen A1-S1-V1 to be associated with the trauma (Pattern 1) and A2-S2-V2 as a control (Pattern 2). The model receives other three inputs: the trauma unit, which is connected to the amygdala, the recalling unit, which is connected to the hippocampus, and the safety/eye movement unit, which represents the input to the PFC during the PE or EMDR therapy.

To model trauma establishment and the subsequent PTSD, we used a protocol consisting of 35 trials (Section 3.1). First, the baseline activation of the hippocampus and amygdala were measured with a single trial of stimulation (Trial 1; one trial lasted 104 time steps; an interval of 104 time steps separated the trials). In trial 1, we activated the input to V1 or the input to V2 without the other elements of the patterns. During the PTSD establishment (Trial 2, corresponding to 3 · 104 timesteps), we activated the whole Pattern 1 together with the trauma binary unit, or Pattern 2 without the trauma input. For the remaining trials (each lasting 104 timesteps) we stimulated V1 or V2 to observe if a single cue was able to activate (a) the amygdala (PTSD establishment), and (b) the hippocampus and the other units of the pattern (memory acquisition).

The simulation of the increased vmPFC excitability in Section 3.2 was obtained by halving the firing threshold θ of the unit. The experience of an event with mild emotional activation was simulated in Section 3.2 by reducing the activation of the unit Trauma (refer to Figure 1) from 1 to 0.3.

To model PE or EMDR therapy (Sections 3.3, 3.4), we first established PTSD as aforementioned. Then we activated the recalling unit projecting to the hippocampal unit associated with the memory trace from trial 11. At the same time, we activated the PFC unit to represent a safety signal delivered to the vmPFC in case of PE, or the eye movement-induced activation of dlPFC in case of EMDR. This protocol corresponds to the reactivation of the traumatic memories during the therapy (imaginal exposure or recalling), associated with the recruitment of the respective cortical areas recruited by the PE and the EMDR (refer to Section 1). After each therapy trial, we delivered a test trial, with only the V1 activation while stopping learning, to measure the PE or EMDR trial-by-trial effect on the cue-induced traumatic memory reactivation. Note that in the figures in Sections 3.3, 3.4, we only show the test trials (V1 activation) after each therapy trial (hippocampus plus PFC activation).

We fitted the data from Nijdam et al. (2012) through a grid search of the parameters ϕ and ψ (Section 2.4). The test reported in Nijdam et al. (2012) compared the timing of symptoms decline in PTSD patients treated with brief eclectic psychotherapy (consisting of session 1 of psychoeducation, sessions from 2 to 6 of imaginal exposure, and the following sessions of cognitive therapy) with that of patients treated with EMDR. Through the measurement of the “Impact of Events Scale - Revised” score (IES-R), they showed a session-by-session decline of symptoms in the two groups of patients. We took the average group data from sessions 2 to 6, where imaginal exposure was administrated to the patients under brief eclectic psychotherapy, and compared them to the corresponding sessions in the EMDR group. The IES-R was normalized to the maximum value and the values were re-scaled, subtracting the IES-R baseline reached after the whole therapy. In order to reproduce the patients' curves of symptom remission after PE and EMDR in Nijdam et al. (2012) we leveraged the fact that the activation of the amygdala is significantly correlated with PTSD symptoms severity (Rauch et al., 2000; Pissiota et al., 2002; Fredrikson and Furmark, 2003; Shin et al., 2004; Protopopescu et al., 2005; Ganzel et al., 2007; Brohawn et al., 2010; Jacques et al., 2011; Peres et al., 2011; McLaughlin et al., 2014; Thomaes et al., 2014; King et al., 2016; Neumeister et al., 2017; Stevens et al., 2017) to reproduce, with the activation of the amygdala unit in our model, the curves of symptom remission after the PE and EMDR in Nijdam et al. (2012) patients. The parameters ϕ and ψ were allowed to vary respectively in steps of 0.05 and 0.5 and ranges of 0.5–2.0 and 0.5–8.5. We measured the distance of the simulations from the real data with the root-mean-squared error (RMSE; Granato and Baldassarre, 2021).

## 3. Results

### 3.1. Trauma Establishment

**Figure 2:**
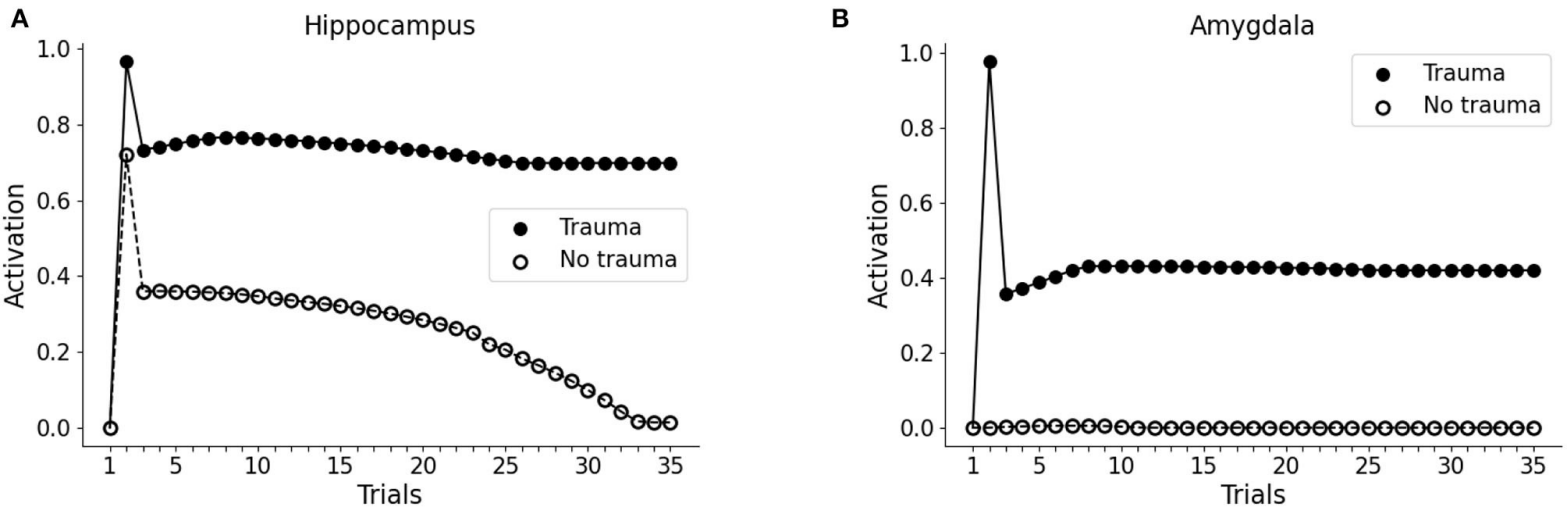
Effect of trauma establishment in hippocampus and amygdala, compared to the control. In the first trial, the baseline activity of the hippocampus **(A)** and amygdala **(B)** were measured during the stimulation of the V1 or the V2 sensory units. In trial 2, pattern 1 (A1-S1-V1) was coactivated with the trauma unit; as a control, pattern 2 (A2-S2-V2) was activated with the trauma unit turned off. During trial 3 and succeeding trials, V1 and V2 were repeatedly activated to investigate the dynamics of the hippocampus and amygdala response. After memory establishment in trial 2, V2 stimulation induces the activation of the hippocampus (**A**, white dots, trials 3–30), but not of the amygdala (**B**, white dots, trials 3-35). On the other hand, the trauma-associated stimulus V1 induces both hippocampus (**A**, black dots, trials 3–35) and amygdala (**B**, black dots, trials 3–35). Over time, the non-traumatic memory trace is lost (**A**, white dots, trial 35), while traumatic associated stimulus V1 is persistently capable to activate the hippocampus (**A**, black dots, trial 35) and amygdala (**B**, black dots, trial 35).

During the first trial of the protocol, we activated the input unit to V1 or V2 only, representing two different visual cues to the visual cortex. In both cases, we observed no activity in the hippocampus or the amygdala (trial 1 in Figures 2A,B). In the second trial, the whole pattern 1 (traumatic pattern: A1-S1-V1 + trauma) or the whole pattern 2 (control pathway: A2-S2-V2) was activated. In this case, the hippocampus firing is induced for both patterns, whereas the amygdala firing is caused only by pattern 1, as a consequence of the activation of the trauma input unit (trial 2 in Figures 2A,B). Note that we observed hippocampal activity only in one unit per pattern, as we show below. During the following trials (trials 3 to 35, Figures 2A,B), we activated only the sensory units V1 or V2.

**Figure 3:**
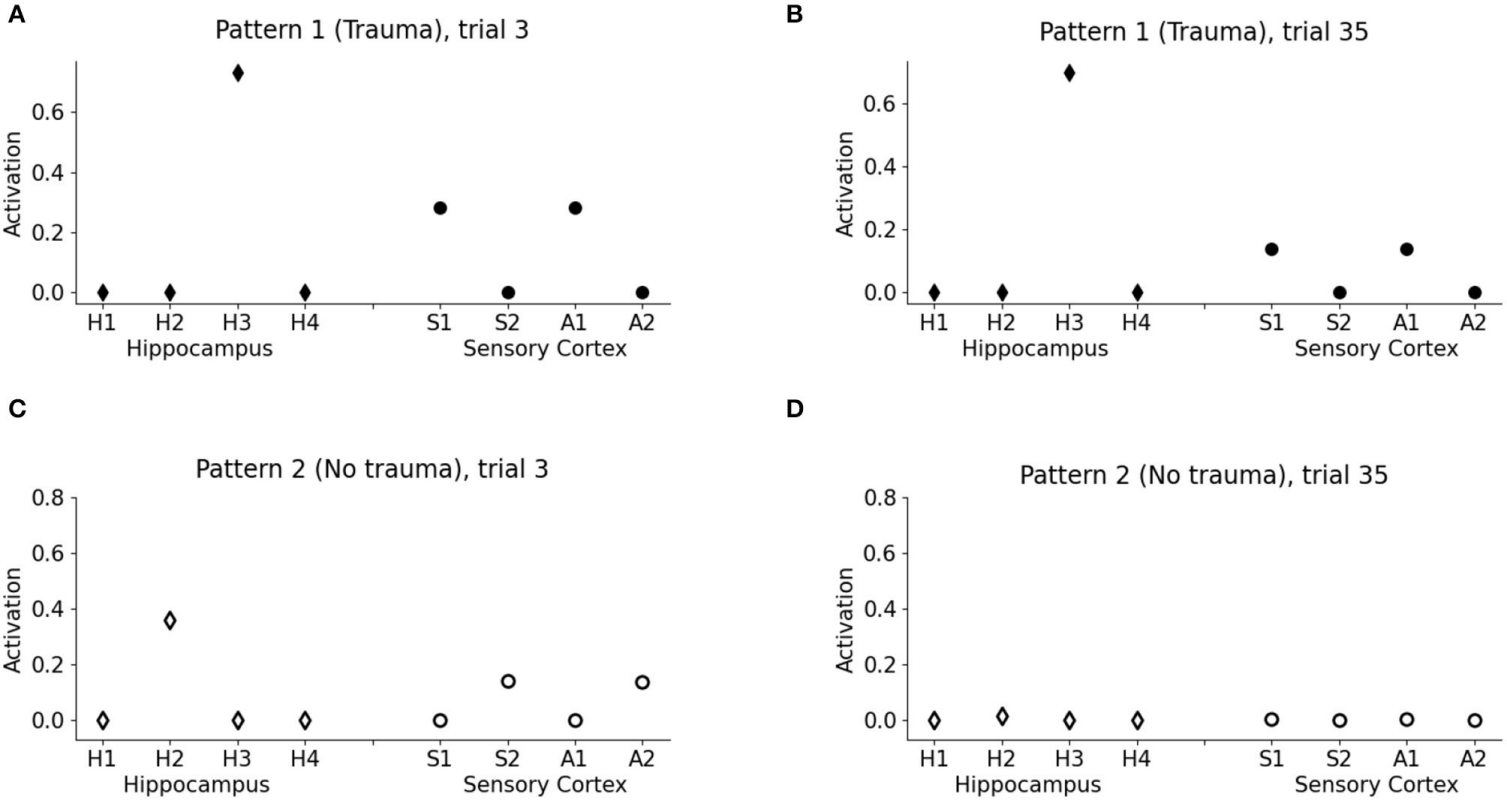
Model memory formation in PTSD and control conditions. **(A)** The graph corresponds to trial 3 of Figure 2 in the condition with the trauma. V1 activates the hippocampal unit H3 and the cortical units S1 and A1 encoding the other elements of pattern 1. **(B)** The graph corresponds to trial 35 of Figure 2 in the trauma condition. The memory trace continues to be activated by V1 even after many trials. **(C)** The graph corresponds to trial 3 of Figure 2 in the control condition with no trauma. V2 activates H2, a different unit of the hippocampus with respect to V1, and also the other cortical elements of the pattern, S2 and A2, although to a lesser extent with respect to PTSD [note the different scales in **(A,C)**]. **(D)** The graph corresponds to trial 35 of Figure 2 in the control condition. After 35 trials, the memory trace is lost and V1 does not elicit activations (the memory has been transferred to the cortex).

Because of the winner-take-all architecture described in Materials and methods, the patterns 1 and 2 activate different units in the hippocampus (Figures 3A,C). While V2 induces an hippocampal activation that slowly fades away during subsequent trials (Figure 2A, white dots: compare trial 3 with trial 35), V1 induces a robust and persistent firing in both hippocampus and amygdala (Figures 2A,B, black dots). This indicates that the initially neutral cue V1 became associated with a threat, causing a persistent amygdala activation.

One of the principal features of PTSD is the emotional flashbacks induced by a trauma reminder. We observed that the presentation of V1 after trauma establishment drives the activation of the hippocampus and also of the other sensory cortices, reinstating the whole pattern 1 (traumatic flashback; Figure 3A). This reinstatement effect is persistent even after several trials (Figure 3B). Instead, the presentation of V2 weakly activates another hippocampal unit (corresponding to a different hippocampal engram) and transiently reinstates the memory trace in the cortex (pattern completion, with the activation of S2 and A2; Figure 3C). However, the memory retrieval induced by V2 is not emotionally loaded (there is no amygdala activation, Figure 2A) and is lost after some trials (Figure 3D).

### 3.2. Differences Between PTSD, Pavlovian Fear, and Emotional Memory

**Figure 4:**
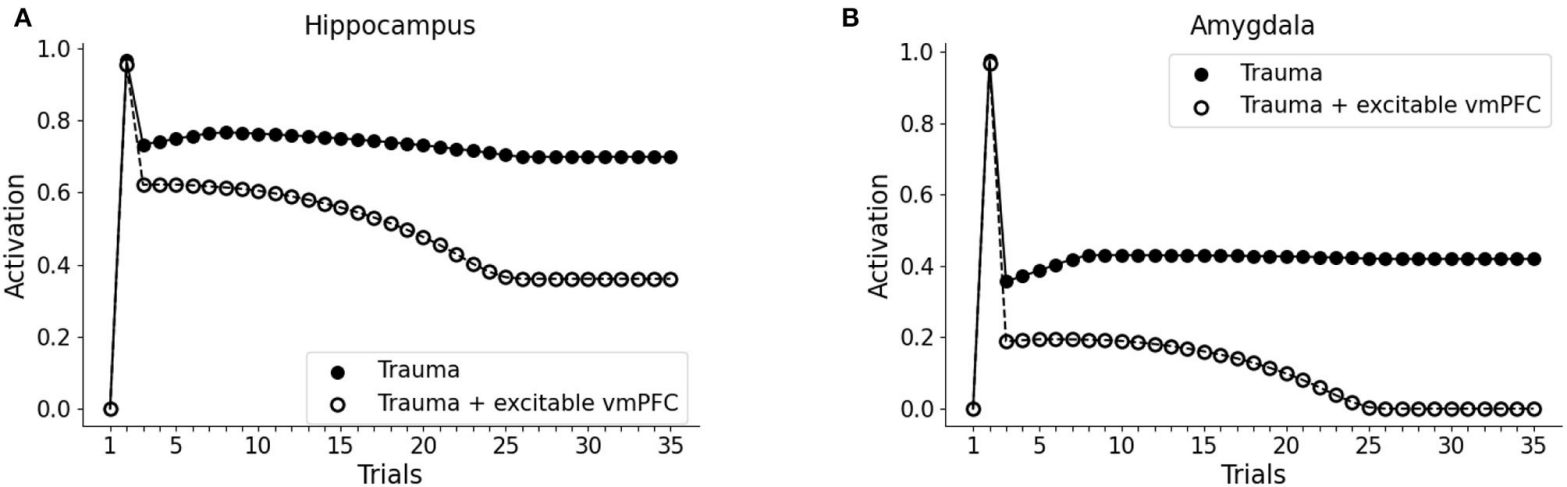
Effect of the high excitability of vmPFC on the triggering of post-traumatic stress disorder (PTSD). Hippocampus **(A)** and amygdala **(B)** activity before (trial 1: V1 input), during (trial 2: V1-A1-S1-Trauma input) and after (trial 3–35: V1 input) trauma, in a model where the vmPFC firing threshold is halved (white dots) compared to the model shown in Figure 2 acquiring the PTSD (black dots).

It has been hypothesized that reduced recruitment of vmPFC is a risk factor for developing PTSD while its higher activation might protect from it (refer to Section 1; Milad et al., 2009; Rougemont-Bücking et al., 2011). We, thus, investigated whether an increase in the excitability of vmPFC would protect the model from developing a persistent activation of the amygdala after the exposure to a traumatic event. We observed that, with a more excitable vmPFC induced by halving its activation threshold (refer to Sections 2, 2.4, for details), the V1 cue still induces amygdala unit activation (trial 3). However, similar to a fear extinction protocol (Mattera et al., 2020), after some trials of exposure to V1 the amygdala unit ceases to activate while the hippocampus remains active even at trial 35 (Figures 4A,B). Thus, the model where the vmPFC is less excitable is predisposed to develop PTSD after a traumatic experience while the model where the vmPFC is more easily recruited is resilient to PTSD and shows a spontaneous fear extinction with exposure to stimuli associated with the trauma.

**Figure 5:**
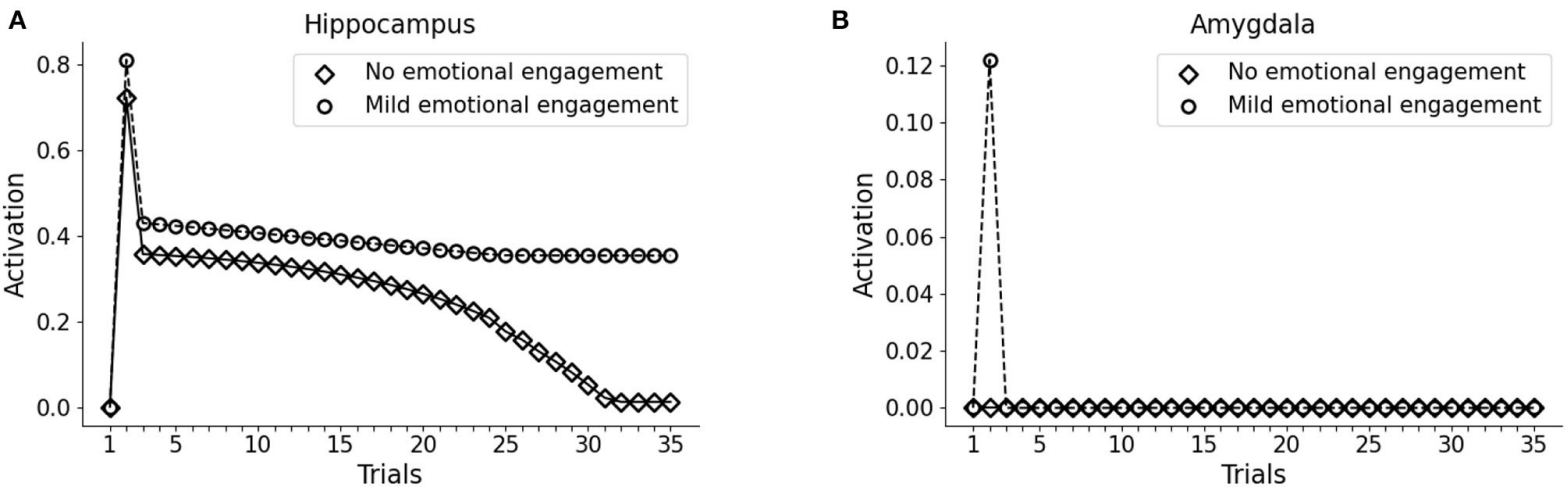
Effect a mild emotional engagement on memory retention. Hippocampus **(A)** and amygdala **(B)** activity with an activation protocol as in Figure 2, except that the vmPFC firing threshold is halved (model robust with respect to PTSD) and that during the second trial the emotional (Trauma) unit is weakly activated (1/10 of activation) to represent a mild (non-traumatic) emotional engagement of the amygdala.

As observed in Figures 2A, 3D (Section 3.1), the model does not establish a long term memory of the elements of the pattern if the emotional (Trauma) unit is not activated. In humans and mice, the longevity of memory traces depends on the emotional charge associated with experiences to be encoded (Hamann et al., 1999; Akirav and Richter-Levin, 2002; Kilpatrick and Cahill, 2003; Huff and Rudy, 2004; Phelps et al., 2004; Huff et al., 2006; Roozendaal et al., 2009; Murty et al., 2011; Hansen, 2017). We, thus, reasoned that in the model a weak amygdala activation during the experience of a sensory pattern (V1-A1-S1) would induce a subsequent enhancement of memory retention. This might correspond to a mild emotional engagement accompanying an experience. To test this, during trial 2 of the protocol we exposed the version of the model seen above, non-predisposed to develop PTSD (high vmPFC excitability), to the pattern V1-A1-S1, together with an activation of 0.1 (instead of 1) of the emotional (Trauma) unit. In this condition, we observed that the hippocampal unit activation persisted throughout all the subsequent trials of V1 presentation, while a pattern that was not associated with the mild amygdala unit activity progressively faded away (Figures 5A,B).

### 3.3. The PE Therapy

**Figure 6:**
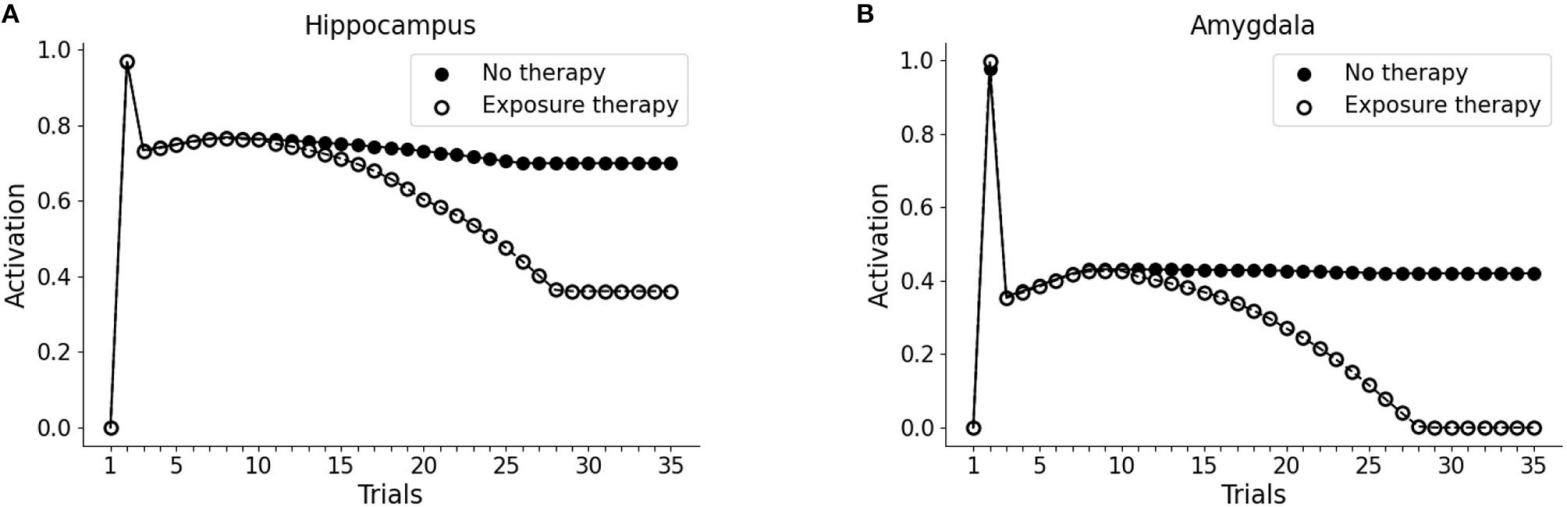
Effect of PE on hippocampus **(A)** and amygdala **(B)**. The first 10 trials were as in Figure 1: trial 1 was the baseline V1 activation of hippocampus and amygdala; trial 2 marked the trauma experience (A1-S1-V1-Trauma); trials 3–10 involved the re-experience of V1 to observe the effect of the trauma. After trial 10, however, we administered PE for 20 sessions (20 trials in the model). PE consisted in the activation of the hippocampal memory trace (unit H3 from the Figures 3A and B), coupled with the activation of vmPFC. Each time point in trials 11–30 corresponds to a V1-activation test after a PE session (trial). After the 20 trials of therapy, hippocampal activation is reduced to a plateau **(A)** and amygdala activation becomes zero **(B)**: the memory has been freed from the negative emotional load.

Next, we investigated whether the PTSD model is capable of trauma symptoms remission through different trials of hippocampal reactivation (mimicking imaginal exposure) coupled with vmPFC activation (mimicking safety signals from the therapy setting). As shown in Figures 6A,B, after the first 10 trials (baseline: trial 1; trauma: trial 2; after-trauma: trials 3–10), we delivered PE therapy for 20 trials (trials 11–30). For each trial of therapy (not shown in figures), we did a subsequent test trial of V1 exposure, to observe the effect of PE on hippocampus and amygdala activation (dots in the figure). PE progressively reduces V1-induced recruitment of the hippocampus (Figure 6A) and amygdala (Figure 6B).

### 3.4. Differences Between the PE and the EMDR Therapies

**Figure 7:**
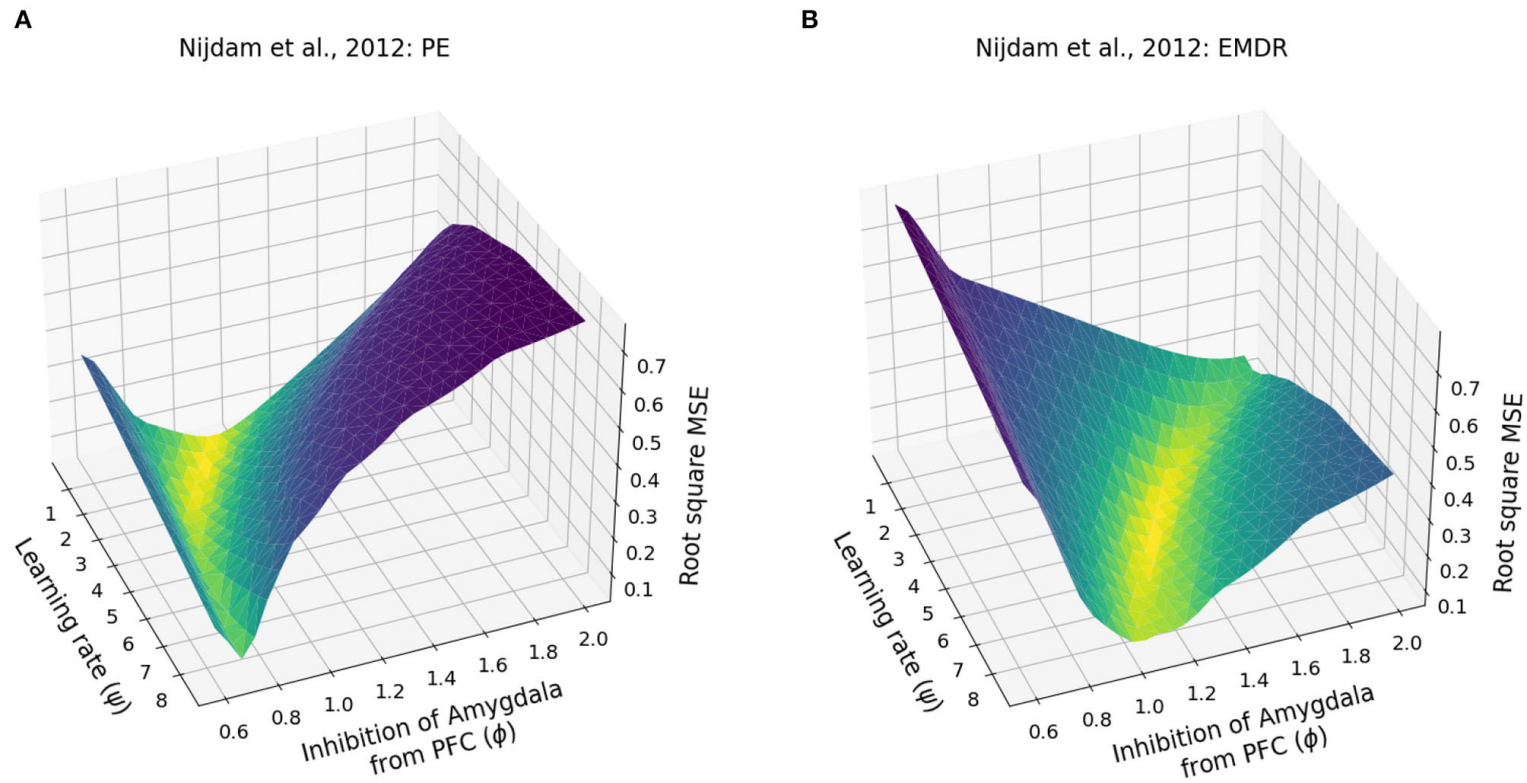
Patients data fitting. **(A)** Root square MSE for the fitting of the PE data from Nijdam et al. (2012). **(B)** Root square MSE for the fitting of the EMDR data from Nijdam et al. (2012).

**Figure 8:**
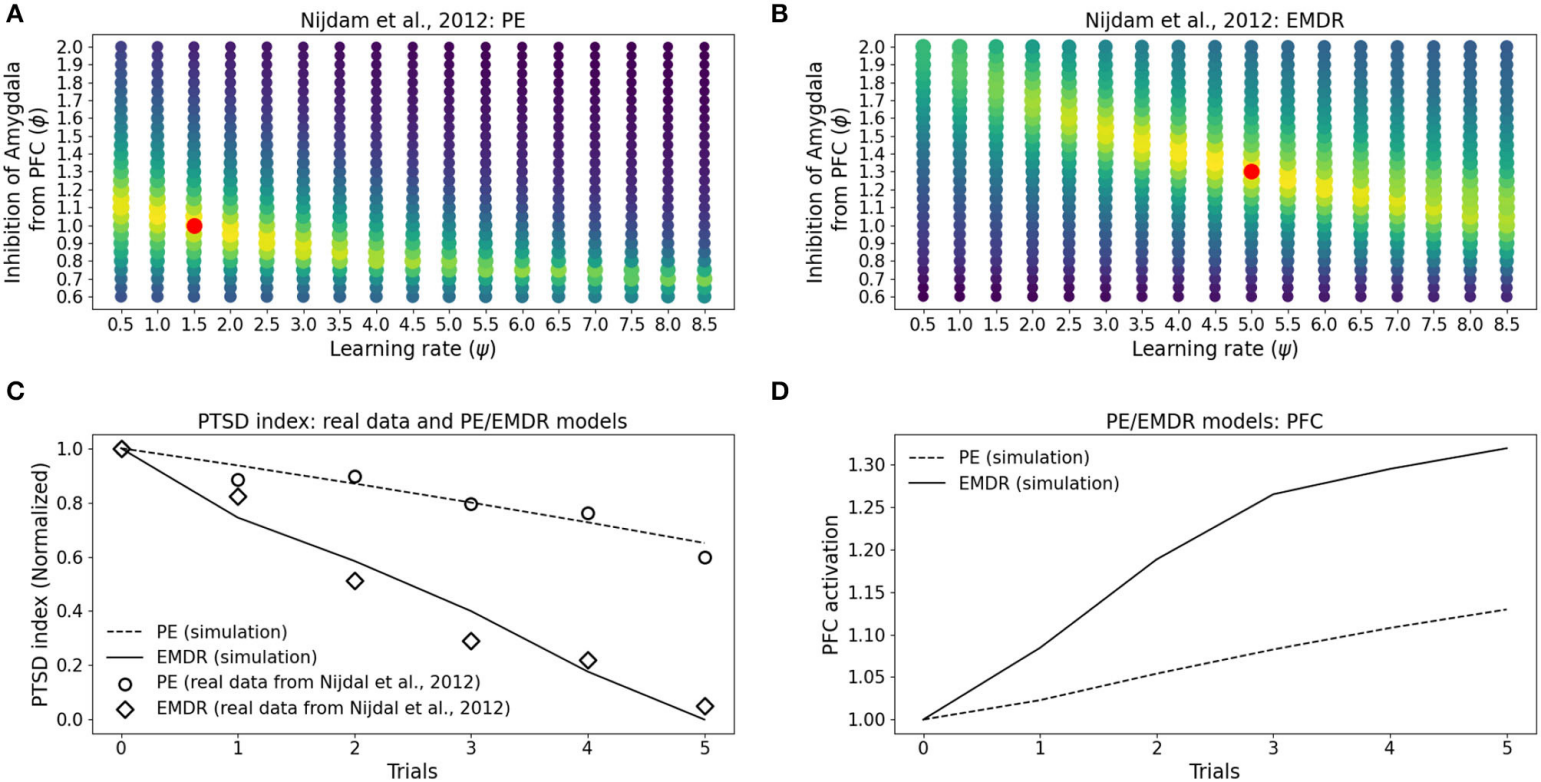
Eye movement desensitization and reprocessing therapy is compared to PE therapy. Each dot in **(A,B)** represents a simulation to fit the data of PE **(A)** and EMDR **(B)** in Nijdam et al. (2012). The size and the color (from blue to yellow) of the dots are proportional to the root square MSE: the best fitting model is marked by a red dot. **(C)** Comparison between the symptoms remission curves obtained with the model, using the parameter combinations indicated by the red dots in graphs **(A)** PE and **(B)** EMDR, and the actual experimental data from Nijdam et al. (2012). For the real data, the PTSD index is represented by the normalized IES-R score (Nijdam et al., 2012; refer to Section 3.4); in the model, the PTSD index represents the normalized amygdala unit activation following the presentation of the reminder cue. **(D)** PFC activation in correspondence to the simulation trials in **(C)**.

Finally, we investigated whether the fast symptom improvement that follows EMDR could be explained by an increase in the cortical learning rate, induced by slow waves, or by a more powerful amygdala inhibition from dlPFC, compared to vmPFC. For this purpose, we modulated the cortical learning rate ψ and the weight ϕ of the inhibitory connection from the PFC to the amygdala to find the best fit of the data from Nijdam et al. (2012). The results of this test show that the set of ϕ and ψ parameters that provide the most accurate fitting of the PE data and EMDR data are well-segregated from other values producing a worse fitting (Figures 7A,B, 8A,B).

In particular, the best combination for PE (ψ = 1.5, ϕ = 1; MSE = 0.039; Figure 8A) and the best combination for EMDR (ψ = 5, ϕ = 1.3; MSE = 0.075; Figure 8B) imply that the cortical learning rate is more than tripled during EMDR with respect to PE, and dlPFC has a 30% higher capacity to inhibit amygdala compared to vmPFC in PE. This results in a faster amygdala deactivation (Figure 8C) and a higher PFC recruitment (Figure 8D) over the course of the therapy sessions.

**Figure 9:**
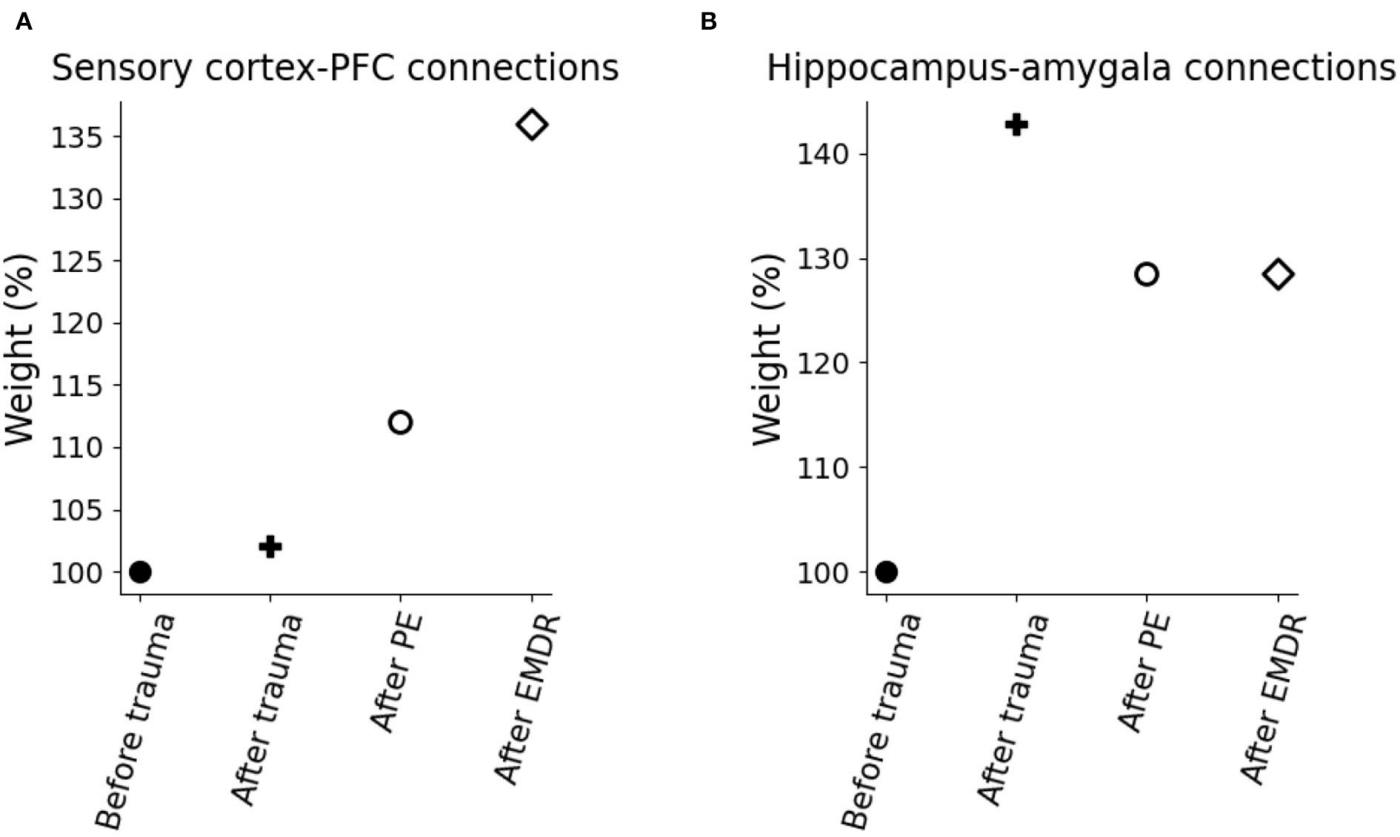
Model weight modification due to trauma and therapy. **(A)** Connection weights linking the sensory cortices to the PFC. **(B)** Connection weights linking the hippocampus to the amygdala.

To investigate the mechanisms of trauma establishment and extinction in PE and EMDR, we analyzed the modifications of the connection weights after trauma and PE or EMDR therapy (Figures 8A,B). After trauma, the connection from the hippocampus to the amygdala is potentiated (Figure 8B), while the sensory connections to the vmPFC and dlPFC are almost unchanged (Figure 8A). Both therapies cause LTP onto the sensory-prefrontal connections (Figure 8A) and LTD onto the hippocampus-amygdala connections (Figure 8B). However, compared to PE, EMDR induces a stronger cortical connection strengthening, and this results in a higher PFC activation (Figure 9A) and a faster amygdala inhibition (Figure 9B).

## 4. Discussion

### 4.1. Contribution of the Model to PTSD Understanding

This study has presented a model capable of recapitulating a core symptom of PTSD, namely the emotional flashbacks enacted by a trauma-reminding cue and the consequent amygdala hyperactivation. An initial neutral cue can be associated with other neutral cues through the sensory-cortex-to-hippocampus and hippocampus-to-sensory-cortex plastic connections. Then, the representation of one of the cues can reactivate the other cortices through the hippocampal hub (Figures 2A, 3C), similarly to the associative cortex-hippocampus network in Alvarez and Squire (1994).

Following previous theoretical predictions (Tryon, 1998; Radell et al., 2017), we showed in Figures 3A,B how a bidirectional associative neural network can indeed be used to model the traumatic flashback in PTSD. Indeed, after a pattern of cues has been temporally coupled with the amygdala activation, a reminding cue reinstates not only different associated elements encoded in other cortices, but also activates the amygdala (Figure 2B). This is in line with data reporting amygdala activation in patients with PTSD exposed to trauma-related stimuli (Liberzon et al., 1999; Pissiota et al., 2002; Shin et al., 2004; Protopopescu et al., 2005).

On the other hand, a pattern of stimuli non-associated with the emotional activation of the amygdala is not retained for long in the hippocampus (Figures 2A, 3C,D). This is coherent with the well-known role of the amygdala in the modulation of synaptic plasticity and memory formation in the hippocampus, both in rodents and in humans (Hamann et al., 1999; Akirav and Richter-Levin, 2002; Kilpatrick and Cahill, 2003; Huff and Rudy, 2004; Phelps et al., 2004; Huff et al., 2006; Roozendaal et al., 2009; Murty et al., 2011). Interestingly, it has been shown that, besides fear neurons, the murine amygdala contains reward neurons activated by positive experiences (Kim et al., 2016; Zhang and Li, 2018; Lutas et al., 2019). These neurons send projections to the hippocampus and potentiate spatial memory consolidation (Yang et al., 2016; Yang and Wang, 2017). Together, these observations contribute to the idea that the amygdala, as well as other brain areas associated with a positive or negative emotional content (e.g., locus coeruleus; Hansen, 2017) signal salient memories to be retained in the hippocampus, as also shown in our simulations (Figures 5A,B). The mechanism of amygdala-induced memory consolidation described above, however, can become maladaptive and lead to PTSD. In our model, the ability of PFC to recruit the amygdala inhibiting areas determines if a traumatic memory will be spontaneously extinguished by the exposure to a trauma-reminding cue or not (Figures 4A,B). In particular, when PFC is not sufficiently intrinsically excitable for a spontaneous trauma remission, the exposure must be paired with an external activation of the PFC, represented in our model by a “safety” input (Figure 1) and in real patients by the therapeutic setting. Indeed, feeling safe during the imaginal reliving of traumatic memories is an important component of the therapy, and it has been suggested that patients gradually learn to incorporate safety information into the traumatic memories (Rothbaum and Schwartz, 2002). Coherently with this view, over the course of the PE sessions, our model learns to autonomously recruit the PFC when the trauma-reminding cue is presented (refer to PE simulation in Figure 8D). This relies on an increase in the strength of the connections between the sensory areas and the PFC (Figure 9A).

While a high percentage of the population is exposed to traumatic events, PTSD only occurs in a subgroup (Alisic et al., 2014; Musazzi et al., 2018) of people for which the symptoms can last even 40 years (Orr et al., 1993). Three main hypotheses, each supported by some experimental findings, have been proposed to explain the susceptibility to the trauma and its long term persistence (Careaga et al., 2016). One hypothesis suggests that some individuals are predisposed to PTSD because of a higher conditionability, meaning that the association between the conditioned stimulus and the unconditioned stimulus is acquired more strongly than in the healthy population (Orr et al., 2000; Blechert et al., 2007; but refer to Milad et al., 2008). A second hypothesis proposes that, in patients with PTSD, the distressing conditioned responses acquire themselves the role of unconditioned stimuli, resulting in a self-strengthening cycle. This predicts a fear incubation effect (Eysenck, 1982), where the conditioned fear responses would become greater over time with the repeated presentation of the trauma-reminding cues, instead of being extinguished (Sandin and Chorot, 2002; but see Nicholaichuk et al., 1982). A third hypothesis postulates a defective extinction (Davis et al., 2000), supported by the fact that patients with PTSD, compared to healthy controls, show deficits in extinction learning and recall in Pavlovian fear paradigms (Blechert et al., 2007; Milad et al., 2008).

In our model, the PFC excitability determines the susceptibility to the trauma. We did not find any difference in the conditionability when the models with hypo- and hyper-excitable PFC were confronted (Figures 4A,B), thus supporting the experimental findings of Milad et al. (2008) rather than those of Orr et al. (2000). Instead, the model shows that reduced PFC recruitment results in defective extinction (Figures 4A,B). Coherently with these results, it has been observed that extinction retention in a protocol of Pavlovian fear and the vmPFC activation are correlated and that patients with PTSD are defective in both (Milad et al., 2009).

Finally, we found a fear incubation effect in the model (refer to trials 3–8 in Figure 2B), where the presentation of the trauma-related cue V1, without the trauma unit activation, induces further conditioning. This occurs because, in the model, the association between the memory trace and the trauma resides in the strength of the connection between the hippocampus and the amygdala (Figure 9B), as suggested by research in murine (contextual fear conditioning; Kim and Cho, 2020) and humans (correlation between hippocampus-amygdala functional connectivity and IES-R scores; Li et al., 2017). In the absence of sufficient inhibition from the PFC, as in the simulation of Figure 2, the loop between the hippocampus and amygdala (Figure 1) enacts the self-strengthening cycle between the conditioned stimulus and the conditioned response hypothesized by the fear incubation paradigm (Eysenck, 1982).

### 4.2. Contribution of the Model to EMDR Understanding

Despite initial skepticism, due to the discussion about underlying neurobiological mechanisms (Lohr et al., 1998; Herbert et al., 2000), several meta-analyses and international guidelines show that EMDR is an effective treatment for PTSD (INSERM Collective Expertise Centre, 2004; Ursano et al., 2004; Bisson et al., 2007, 2019; World Health Organization, 2013; de Roos et al., 2017; Khan et al., 2018; Lewey et al., 2018; Navarro et al., 2018; Wilson et al., 2018; Karatzias et al., 2019; Bastien et al., 2020; Mavranezouli et al., 2020). Our model allowed us to analyze patients' data in search of the most suitable parameter set that explains the experimental findings. Coherently with the positive correlation found between IES-R measurement of PTSD symptoms and amygdala activation (McLaughlin et al., 2014), we could reproduce the IES-R curves from Nijdam et al. (2012) using the activity of the amygdala as a proxy. The parameters found by the grid search analysis suggest that PE and EMDR make use of different mechanisms to exert their therapeutic effect (Figures 7, 8). While cognitive and exposure therapies are centered on the activities focusing on the traumatic memories, during an EMDR session, the patient is invited to notice the trauma with a distant attitude (“Imagine you are on the train and the scenery is passing by. Just notice the scenery without trying to grab hold of it or make it significant.”; from Shapiro, 1995). It is known that distancing and distraction activate the prefrontal, cingulate, and parietal cortices (among which are the dlPFC) and are very effective in emotion regulation, in particular in amygdala downregulation (McRae et al., 2010; Kanske et al., 2011; Dörfel et al., 2014). Moreover, de Voogd et al. (2018) observed that the dlPFC is activated following bilateral eye stimulation. The parameter ϕ of the model, which in EMDR resulted to be 30% higher than in PE (Figures 8A,B), indicates that: (1) the regions recruited by EMDR are different from the regions recruited by PE; (2) EMDR-recruited regions have a higher capacity to inhibit amygdala compared to the regions activated during PE. The parameter ψ, which is 3.3 times higher in EMDR than PE (Figures 8A,B), indicates an enhanced cortical learning rate during the bilateral stimulation, as suggested by the slow waves recorded during the therapy in patients (Harper et al., 2009; Pagani et al., 2011, 2012). The physiological alternation during sleep between slow waves and rapid eye movement periods promotes memorization and facilitates the elaboration and contextualization of traumatic memories (Carletto et al., 2017). The evidence that EMDR therapy induces the appearance of slow waves concurrently with bilateral stimulation speaks in favor of faster synaptic and neuronal plasticity and hence faster processing of traumatic memories as compared to other psychotherapies (Pagani et al., 2012, 2017).

### 4.3. Limitations

The model reproduces simplified connectomics between sensory cortical areas and the PFC, where direct connections reach the vmPFC and dlPFC from unimodal sensory areas. Indeed, secondary sensory cortices have a role in storing and retrieval of fear memory content (Sacco and Sacchetti, 2010) and PFC receives direct inputs from the whole cortex in murine, primates, and humans (Öngür and Price, 2000; Ährlund-Richter et al., 2019). However, relevant evidence suggests the presence, for fear-related input, of at least a relay station (the anterior cingulate cortex) between secondary cortices and PFC (van Heukelum et al., 2020; Kredlow et al., 2022). Another area not considered in the model, that has been suggested to be involved in PTSD (Yoshii, 2021) and, at least in murine, is the effect of alternating bilateral sensory stimulation (Baek et al., 2019), is the thalamus. The level of detail and the number of brain regions included in the model are a trade-off between biological plausibility and computational complexity, in order to test a large scale hypothesis of the PTSD network and the PE and EMDR mechanisms while maintaining the number of parameters reasonably low (Eliasmith and Trujillo, 2014). This allowed us to avoid a large number of model degrees of freedom as well as to perform a grid search with hundreds of simulations. Future study could include more brain areas in this model in order to investigate their potential role in trauma and therapy.

Two limitations concern the data fitting. First, we did not fit data from the single patients, but only from the average curve reported in the literature (Nijdam et al., 2012). This provided a proof of concept of the putative PE and EMDR mechanisms, but a more robust analysis will be performed in the future on the single patients if the data will be obtainable. This would test the robustness of the conclusions drawn from the present study on a dataset containing individual participant data. Moreover, it would allow the use of the model as a tool to investigate the individual differences in trauma remission, response to the therapy, and PTSD susceptibility factors. Second, we used the amygdala activation as a proxy of patient symptoms, measured in the original work with the IES-R. Although several experiments have found a linear correlation between the symptoms severity and the amygdala recruitment (refer to Section 1; in particular, for the IES-R refer to McLaughlin et al., 2014), the best solution would be to use the amygdala activation in the model to fit fMRI data from patients. This would require fMRI measurements of the amygdala activation during the exposition to trauma reminders, taken between different therapeutic sessions of EMDR or PE. To our knowledge, these datasets are currently not available in the literature. Despite the technical limitations of the procedure, this would furnish a novel and powerful link between the time course of amygdala deactivation, the time course of symptoms remission during therapy, and the computational modeling of the underlying mechanisms.

## Data Availability Statement

The original contributions presented in the study are included in the article/Supplementary Material, further inquiries can be directed to the corresponding author/s.

## Author Contributions

AM, MP, and GB conceived the model and wrote this article. AM and AC conducted the simulation. AM, AC, GG, MP, and GB analyzed the results. All authors reviewed the article and approved the submitted version.

## Funding

This project has received funding from the European Union's Horizon 2020 Research and Innovation Programme under Grant Agreement No. 713010 (GOAL-Robots—Goal-based Open-ended Autonomous Learning Robots) and a dedicated Grant from EMDR Italy Association.

## Conflict of Interest

The authors declare that the research was conducted in the absence of any commercial or financial relationships that could be construed as a potential conflict of interest.

## Publisher's Note

All claims expressed in this article are solely those of the authors and do not necessarily represent those of their affiliated organizations, or those of the publisher, the editors and the reviewers. Any product that may be evaluated in this article, or claim that may be made by its manufacturer, is not guaranteed or endorsed by the publisher.
