# Supplementary material for: A Biologically Inspired Neural Network Model to Gain Insight Into the Mechanisms of Post-Traumatic Stress Disorder and Eye Movement Desensitization and Reprocessing Therapy
Source: Front Psychol. 2022 Jul 13;13:944838. doi: 10.3389/fpsyg.2022.944838 (PMC9326218; doi:10.3389/fpsyg.2022.944838)
Supplement: Supplementary file 1 [file Table_1.pdf]

Supplementary Material

1 SUPPLEMENTARY TABLE

Table S1. Parameters of the model

Parameter	Value
τ	10
Binary inputs	1
Trauma input (in figure 5)	0.1
θ (sensory cortex)	0
θ (hippocampus, amygdala, PFC)	0.18
θ (vmPFC in figure 4)	0.09
α (cortico-cortical connections)	1^{-6}
α (other connections)	1^{-5}
ρ (amygdala, PFC)	0.2
ρ (others)	0.35
Weight (input to sensory)	2
Weight (sensory to sensory)	-3
Weight (sensory to PFC)	0.2 (min = 0.2; max = 0.5)
Weight (sensory to hippocampus)	0.2-0.4 (min = 0.2; max = 0.4)
Weight (hippocampus to sensory)	0.2-0.4 (min = 0.2; max = 0.5)
Weight (hippocampus to hippocampus)	-3
Weight (hippocampus to amygdala)	0.7 (min = 0.7; max = 1)
Weight (amygdala to hippocampus)	1
ϕ (PFC to amygdala, figures 2-6)	-1
